# Normal Gun-Deafness

**Published:** 1918-08

**Authors:** 


***Normal Gun Deafness.*** By T. B. Jobson. *The Lancet*, No. 4911, Vol. CXCIII.

To get anything like correct results with tuning fork tests the patient must concentrate on the subject and help the investigator all he can. After trying forks of different wave-lengths the author decided to use C2, which gives 512 double vibrations. The higher forks are heard for such short periods that no accurate results could be obtained in an investigation of this kind. The following are the details of the cases examined :

Total examined : 73.

13 cases were excluded for the following reasons :

| | |
|---|---|
| For old suppurative otitis media | 8 |
| Nasal obstruction | 1 |
| Enlarged tonsils | 2 |
| Head injury | 1 |
| Otorrhea | 1 |

The conditions of the membranes in the 60 accepted cases were as follows :

| | |
|---|---|
| Normal | 39 |
| Dull | 16 |
| Retracted | 3 |
| Increased mobility | 2 |
| Cerumen removed | 2 |

The type of deafness is a fairly definite one — mixed obstructive and nerve deafness.

From this investigation it may be concluded that exposure to gun-fire in the present war often produces rapidly a permanent deafness. The amount of deafness as shown by a C2 fork is about 10 seconds aerial conduction and 4 seconds of bone conduction.

